# Protein Kinase C Overexpression Suppresses Calcineurin-Associated Defects in *Aspergillus nidulans* and Is Involved in Mitochondrial Function

**DOI:** 10.1371/journal.pone.0104792

**Published:** 2014-08-25

**Authors:** Ana Cristina Colabardini, Laure Nicolas Annick Ries, Neil Andrew Brown, Marcela Savoldi, Taísa Magnani Dinamarco, Marcia Regina von Zeska, Maria Helena S. Goldman, Gustavo Henrique Goldman

**Affiliations:** 1 Laboratório Nacional de Ciência e Tecnologia do Bioetanol – CTBE, Campinas, São Paulo, Brazil; 2 Faculdade de Ciências Farmacêuticas de Ribeirão Preto, Universidade de São Paulo, São Paulo, Brazil; 3 Faculdade de Filosofia, Ciências e Letras de Ribeirão Preto, Universidade de São Paulo, São Paulo, Brazil; Georg-August-University of Göttingen Institute of Microbiology & Genetics, Germany

## Abstract

In filamentous fungi, intracellular signaling pathways which are mediated by changing calcium levels and/or by activated protein kinase C (Pkc), control fungal adaptation to external stimuli. A rise in intracellular Ca^2+^ levels activates calcineurin subunit A (CnaA), which regulates cellular calcium homeostasis among other processes. Pkc is primarily involved in maintaining cell wall integrity (CWI) in response to different environmental stresses. Cross-talk between the Ca^2+^ and Pkc-mediated pathways has mainly been described in *Saccharomyces cerevisiae* and in a few other filamentous fungi. The presented study describes a genetic interaction between CnaA and PkcA in the filamentous fungus *Aspergillus nidulans*. Overexpression of *pkcA* partially rescues the phenotypes caused by a *cnaA* deletion. Furthermore, CnaA appears to affect the regulation of a mitogen-activated kinase, MpkA, involved in the CWI pathway. Reversely, PkcA is involved in controlling intracellular calcium homeostasis, as was confirmed by microarray analysis. Furthermore, overexpression of *pkcA* in a *cnaA* deletion background restores mitochondrial number and function. In conclusion, PkcA and CnaA-mediated signaling appear to share common targets, one of which appears to be MpkA of the CWI pathway. Both pathways also regulate components involved in mitochondrial biogenesis and function. This study describes targets for PkcA and CnaA-signaling pathways in an *A. nidulans* and identifies a novel interaction of both pathways in the regulation of cellular respiration.

## Introduction

Cellular responses to environmental stimuli are often mediated through G-proteins, which consist of a G-protein coupled receptor (GPCR) and the associated heterotrimeric G-proteins [1]. One such G-protein is phospholipase C which produces the second messengers diacylglycerol (DAG) and inositol 1,4,5-triphosphate (IP_3_) from the cell membrane phospholipid phosphatidylinositol 4,5-bisphosphate. These second messengers subsequently cause an increase in intracellular Ca^2+^ levels [2]. The concentration of intracellular calcium ions (Ca^2+^) serves as a signal for the regulation of many cellular processes and is constantly altered in response to environmental cues and physiological signals [3]. In mammalian cells, a rise in intracellular Ca^2+^ levels causes the activation of the calcineurin phosphatase and the protein kinase C (Pkc) pathways [2]. Protein kinases and phosphatases act as key regulators of signal transduction by adding or removing phosphate groups to their protein targets hence directing the activity, location and function of many proteins [4]. In the filamentous fungus *Aspergillus nidulans*, PkcA was shown to be fundamental to the cell wall integrity (CWI) pathway, is essential for viability, and has been shown to be involved in penicillin production, polarized growth, morphogenesis, septum formation and the apoptosis [5]–[9]. Crosstalk between Pkc and the unfolded protein response (UPR) or other MAP (mitogen-activated protein) kinase cascades has been observed [10], [11].

The MAP-kinase (MAPK) cascades function via the sequential phosphorylation of three protein kinases (MAPKKK, MAPKK, MAPK) resulting in the activation of a multifunctional MAP kinase [12]. MAP kinase phosphorylation cascades are important for relaying, integrating and amplifying intracellular signals and are crucial signaling components involved in many cellular processes [12]. In *Saccharomyces cerevisiae*, MAP kinases control mating, the cellular response to high environmental osmolarity, pseudohyphal development, sporulation of diploid cells and the maintenance of CWI in response to stresses, such as heat stress and low osmolarity [13]. In *S. cerevisiae*, the CWI pathway is activated when Pkc1p phosphorylates Bck1p, a MAPKKK, which in turn activates the two MAPKKs, Mkk1p and Mkk2p, which then go on to phosphorylate the MAPK Slt2p. Mkk1p/2p and Slt2p regulate the expression of many downstream protein targets such as cell wall proteins and enzymes involved in cell wall biogenesis [14]. In *A. nidulans*, a similar mechanism operates in the activation of the CWI pathway. The orthologues of *S. cerevisiae* Bck1p and Slt2p, in *A. nidulans*, BckA (MAPKKK) and MpkA (MAPK), were shown to function downstream of PkcA and were involved in the suppression of apoptosis during heat stress [11].

*A. nidulans* PkcA contains a long conserved N-terminal regulatory region consisting of three subdomains (CN1, CN2 and CN3), which interact with cell membranes [15]. The CN3 subdomain has high similarity with the calcium-binding domain of mammalian PKCs, but the lack of an aspartate residue dramatically decreases the affinity for this ion [16]. In *S. cerevisiae*, the PkcA homologue, Pkc1p, is activated by the Ras-like GTPase Rho1p [17] which in turn is activated by the cell membrane stress sensors Wsc1p (cell wall integrity and stress response component) [18] and Mid2 (mating induced death). These membrane sensors interact with the Rho1p guanine nucleotide exchange factor Rom2 [19]. In contrast to mammalian and *S. cerevisiae* cells, the mechanism of PkcA activation in *A. nidulans* remains unknown.

In filamentous fungi, intracellular Ca^2+^ levels are essential for the regulation of hyphal morphology (branching) and growth (orientation) [20]–[22]. The two major mediators of Ca^2+^-mediated signaling are the Ca^2+^-binding protein calmodulin (CaM) and the Ca^2+^/calmodulin-dependent calcineurin, a serine/threonine protein phosphatase [23]. Calcineurin consists of a catalytic subunit A and a regulatory subunit B, which through its association renders the catalytic subunit inactive [21]. Upon Ca^2+^ and calmodulin binding, calcineurin subunit A dissociates from the regulatory subunit and becomes active [21]. In filamentous fungi, calcineurin mediates growth, cell morphology, mating, virulence and responses to antifungal drugs [21] [24]–[28]. One of the targets of calcineurin subunit A (CnaA) in *A. nidulans* is the transcription factor CrzA. Upon an increase in intracellular Ca^2+^ levels, CnaA becomes active and dephosphorylates CrzA which subsequently translocates to the nucleus [29]. CrzA regulates the expression of *chsB*, which encode a chitin synthase involved in cell wall synthesis [30], P-type Ca^2+^ ATPase-encoding genes, which are involved in Ca^2+^ efflux, and other genes involved in calcium metabolism [31], thus mediating calcium homeostasis.

Although an increase in intracellular Ca^2+^ levels does not directly activate protein kinase C in fungi, as is the case in mammalian cells, there is considerable crosstalk between Ca^2+^-signaling pathways and Pkc-mediated signaling. In *S. cerevisiae*, transcriptional regulation of *FKS2*, which encodes the catalytic subunit of glucan synthase, is governed by a combination of signals mediated by Pkc1p, calcineurin and the Kss1p MAPK pathway components Ste11p and Ste12p, in response to different environmental stimuli [32]. Furthermore, constitutively expressed calcineurin can partially suppress the lysing phenotypes caused by *PKC1* mutations [33]. Similarly, in *Candida albicans* and *Cryptococcus neoformans*, calcineurin was shown to induce *FKS1*, which encodes a β-1,3-glucan synthase subunit also regulated by the CWI pathway [33].

The presented study provides further evidence for a crosstalk between the calcium- and PkcA-mediated signaling pathways in *A. nidulans*. Overexpression of *pkcA* in a Δ*cnaA* background partially suppressed the phenotypic effects caused by the *cnaA* deletion. Furthermore, PkcA seemed to be involved in maintaining intracellular calcium homeostasis through controlling the expression of genes encoding mitochondrial components. This work clearly states the involvement of protein kinase C in various calcium-regulated processes in a filamentous fungus.

## Results

### Genetic interaction between *pkcA* and *cnaA*

**Figure 1:**
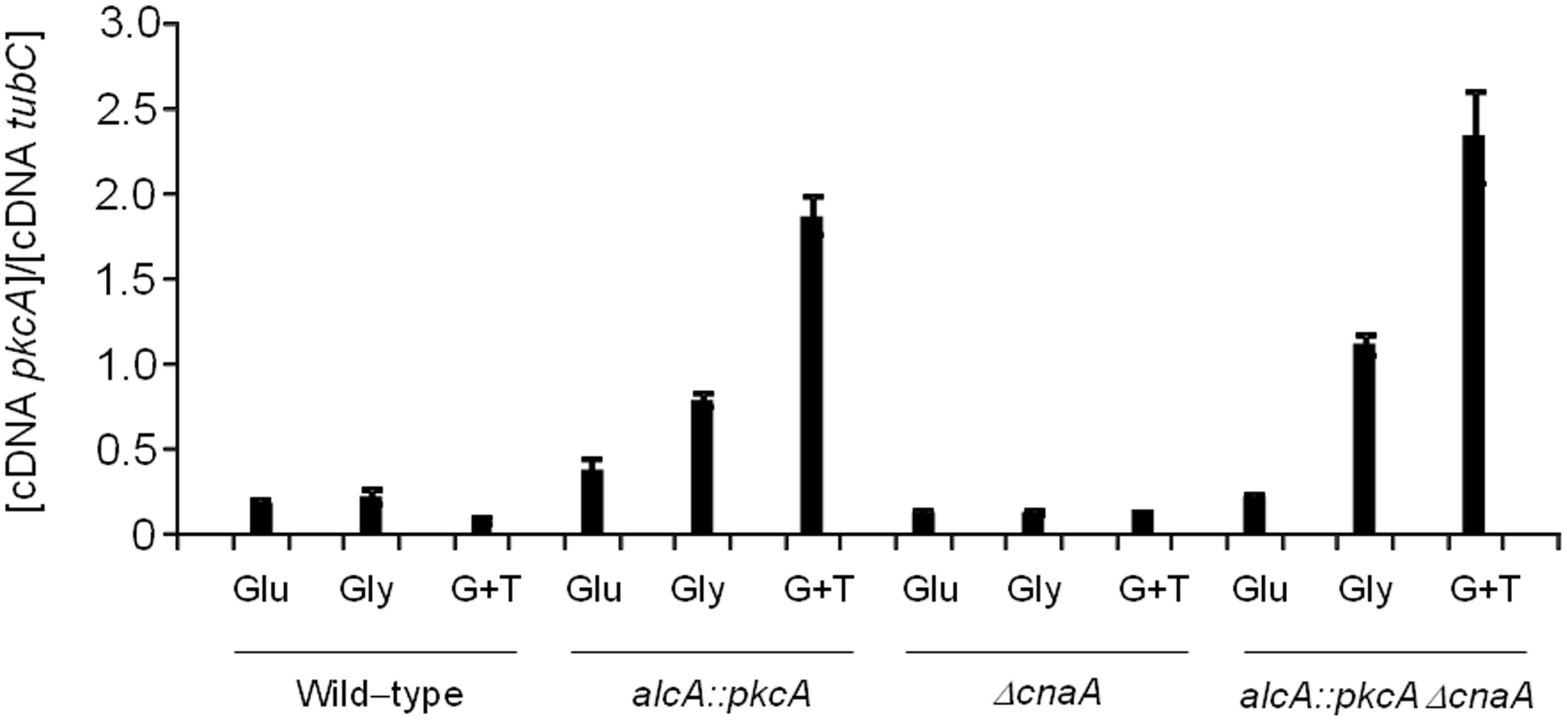
The *alcA::pkcA ΔcnaA* strain has increased *pkcA* mRNA accumulation. The wild−type, *alcA::pkcA*, *ΔcnaA*, and *alcA::pkcA ΔcnaA* strains were grown for 16 hours in minimal medium supplemented either with glucose 2% (G), glycerol 2% (Gly) or glycerol 2% plus 100 mM threonine (G+T), RNA extracted and RT-qPCR performed for *pkcA*.

**Figure 2:**
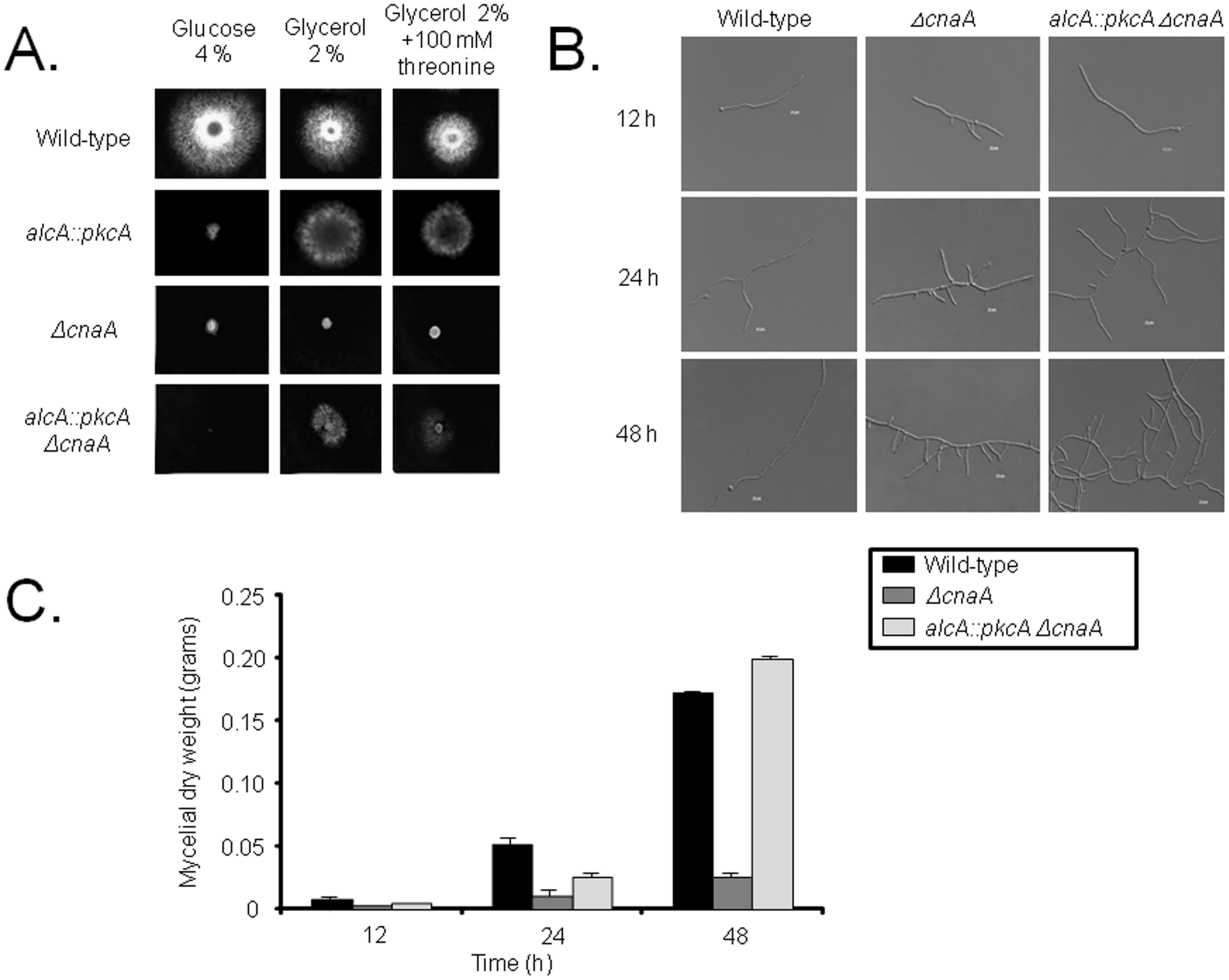
Overexpression of *pkcA* suppresses the phenotype defects caused by the *cnaA* deletion. (A) The wild-type, *alcA::pkcA*, *ΔcnaA* and *alcA::pkcA ΔcnaA* strains were grown for 72 hours at 37°C on agar plates containing either 4% glucose, 2% glycerol or 2% glycerol plus 100 mM threonine, or (B) in liquid media containing 2% glycerol plus 100 mM threonine for 12, 24 and 48 hours at 37°C, before their growth was assessed under the microscope (scale bars indicate 5 µm) and (C) mycelial dry weight measured for three biological replicates from each collected timepoint.

The deletion of the *A. nidulans* calcineurin phosphatase subunit A (CnaA) resulted in severe growth and conidiation defects, increased branching and septation [34], while both PkcA and CnaA are involved in maintaining cell wall integrity [32] [35], [36]. Therefore, a connection between these two proteins may exist. Hence, the *alcA::pkcA ΔcnaA* strain was constructed by sexually crossing an *alcA::pkcA* strain (in which the *pkcA* gene was placed under the regulatory control of the *alcA* promoter) with a *ΔcnaA* strain. Transcription of *alcA* is repressed in the presence of glucose, derepressed in the presence of glycerol and induced to high levels in the presence of ethanol or L-threonine [37]. The *pkcA* mRNA accumulation is increased about 3 to 4−fold when *alcA::pkcA* and *alcA::pkcA ΔcnaA* growth in 2% glycerol+100 mM threonine was compared to glucose 2% for both, respectively (Figure 1). The *alcA*::*pkcA* Δ*cnaA* strain showed a severe growth defect in the presence of glucose when compared to the wild-type strain, and worse than the *ΔcnaA* strain (Figure 2A). Growth of the *alcA*::*pkcA* Δ*cnaA* strain on solid media was partially restored in the presence of glycerol and glycerol plus threonine, when compared to the *ΔcnaA* strain (Figure 2A). The observed phenotypes were confirmed by measuring fungal biomass (dry weight) in liquid media containing 2% glycerol plus 100 mM threonine for 12, 24 and 48 hours at 37°C (Figure 2B). Nevertheless, a relation between branch emergence and septum formation may exist, as increased branching was observed in both the Δ*cnaA* and *alcA*::*pkcA* Δ*cnaA* strains (Figure 2B). After 48 hours of growth in the presence of glycerol plus threonine, the dry weight of *alcA::pkaA ΔcnaA* strain was slightly higher than, while the *ΔcnaA* strain was less than half, of the wild-type strain (Figure 2C).

**Figure 3:**
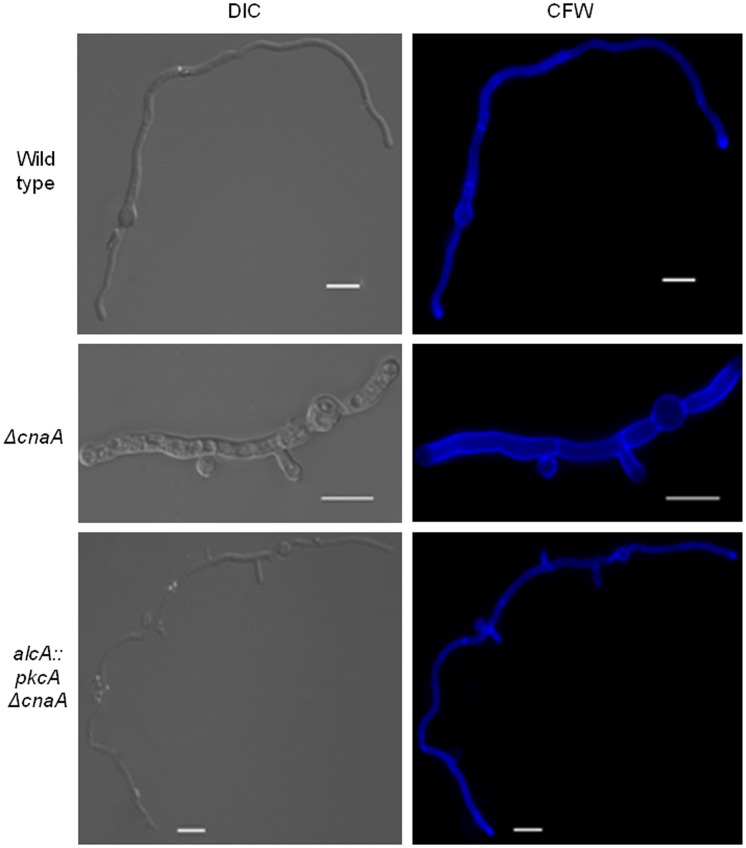
Overexpression of *pkcA* in a *ΔcnaA* background rescues aberrant septa formation in a Δ*cnaA* background. Wild-type, Δ*cnaA* and *alcA*::*pkcA* Δ*cnaA* strains were grown in minimal medium supplemented with 2% (w/v) glycerol plus 100 mM threonine for 16 hours at 30°C before being fixed for 30 minutes at room temperature (RT) and stained with calcofluor white (CFW) for 5 minutes at RT. Mycelial fluorescence was then assessed under the microscope (scale bars indicate 5 µm).

**Figure 4:**
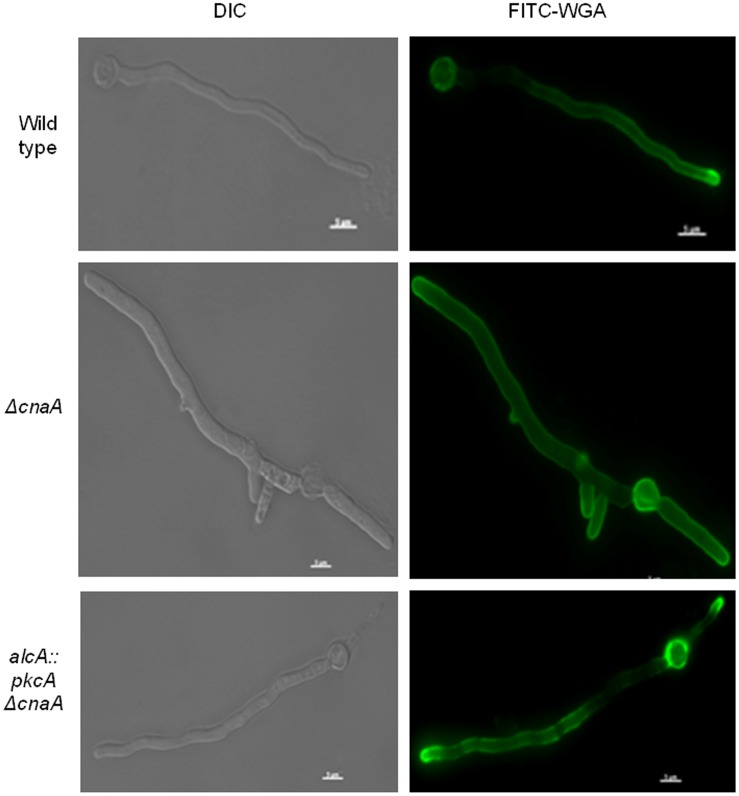
Overexpression of *pkcA* in a *ΔcnaA* background rescues aberrant cell wall deposition in a Δ*cnaA* background. Wild-type, Δ*cnaA* and *alcA*::*pkcA* Δ*cnaA* strains were grown in minimal medium supplemented with 2% (w/v) glycerol plus 100 mM threonine for 16 hours at 30°C before being fixed for 30 minutes at room temperature (RT) and stained with fluorescein isothiocyanate wheat germ agglutinin (FITC-WGA) for 5 minutes at RT. Mycelial fluorescence was then assessed under the microscope (scale bars indicate 5 µm).

As previously observed [34], the *ΔcnaA* germlings contained a higher number of septa (5.9±0.88 septa in 100 germlings) than the wild-type and *alcA*::*pkcA* Δ*cnaA* (1.5±0.53 and 1.1±0.32 septa in 100 germlings) strains. The length between septa of the *ΔcnaA* was shorter than that of the wild− type (Figure 3). The length of the *alcA:: pkcA ΔcnaA* strain was restored under the PkcA-overexpression condition, and the restored length was similar to that of the wild−type (Figure 3). Septa are fungal cell walls which divide the non-apical part of the hypha into symmetrical compartments from which new branches can emerge [38]. One hypothesis is that septa function as a spatial cue for branch emergence in some fungi and that localized spikes in calcium levels may also specify branching sites, although substantial evidence for both theories is lacking [39]. Overexpression of *pkcA* restores the number of septa similar to the wild-type (Figure 3). In *A. nidulans*, FITC (fluorescein isothiocyanate)-conjugated WGA (wheat germ agglutinin) can be used to detect chitin that is deposited into the cell wall of the growing hyphal tip [40]. FITC-WGA staining of actively growing hyphae revealed that in approximately half (46%, 100 germlings; Figure 4) of the Δ*cnaA* hyphae, cell wall extension at the apex was lost when compared to the wild-type strain (100%, 100 germlings, Figure 2B). On the other hand, in the *alcA::pkcA ΔcnaA* strain, cell wall deposition was the same as in the wild-type strain (100%, 100 germlings; Figure 4). These results support the hypothesis of a genetic interaction between the signaling pathways that involve PkcA and CnaA.

### MpkA is targeted by calcineurin-mediated signaling

**Figure 5:**
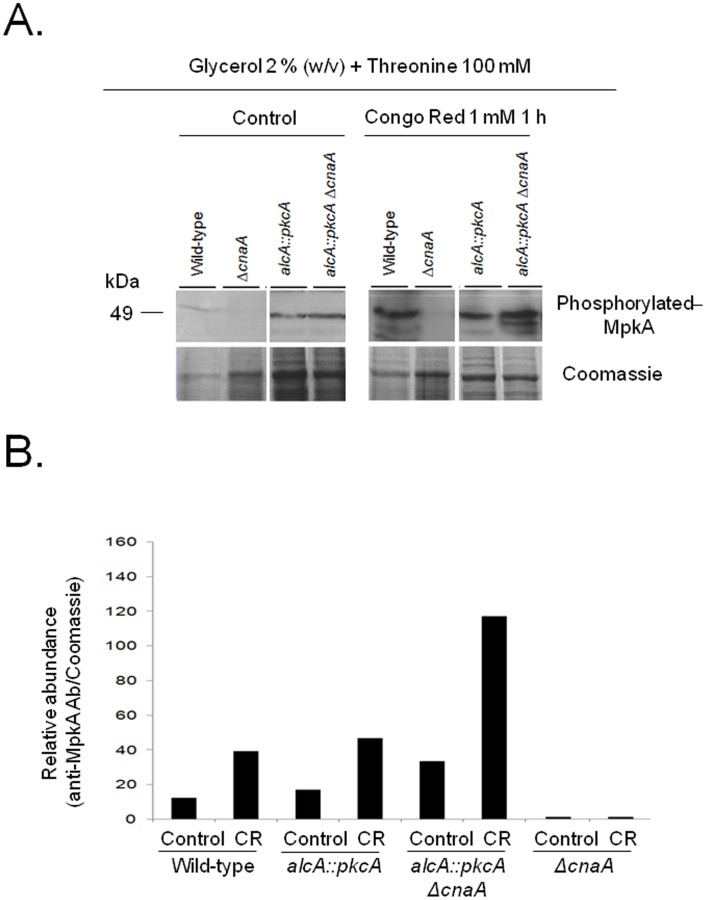
Overexpression of *pkcA* in a Δ*cnaA* background restores the levels of phosphorylated MpkA. Western blots (A) of the protein crude extract from the wild-type, *ΔcnaA*, *alcA::pkcA* and *alcA::pkcA ΔcnaA* strains, when grown in 2% glycerol plus 100 mM threonine for 16 hours at 30°C before being treated with Congo Red (CR) for 1 hour. Phosphorylated MpkA (49 kDa band) was probed for with anti-MpkA antibodies (B) and signal intensities were quantified using the Image J software by dividing the intensity of Western band by a the Coomassie stained protein bands. kDa, kilo Daltons. These results are from a single representative experiment from three different repetitions that provided comparable results.

In *S. cerevisiae*, Pkc1p (the homologue of *A. nidulans* PkcA), is involved in the regulation of cell wall construction through indirectly phosphorylating the MAP kinase Mpk1p, which in turn activates the transcription factor Rlm1p. This transcription factor regulates the expression of genes whose products are important for cell wall biosynthesis [14]. The *A. nidulans rlmA* and *mpkA* are functional orthologs of *S. cerevisiae RLM1* and *MPK1* [41]. Cell wall stress, as experienced during hyphal extension and the addition of Congo red (CR) to liquid cultures, results in the phosphorylation of MpkA [10] [41]. Hence, the MpkA phosphorylation state in the *ΔcnaA* and *alcA::pkcA ΔcnaA* mutant strains was investigated. The wild-type strain contained a basal level of phosphorylated MpkA in the presence of 2% glycerol plus threonine, while the addition of CR resulted in a ∼4-fold increase of MpkA phosphorylation levels (Figures 5A and 5B). In the *ΔcnaA* strain, MpkA phophorylation levels were roughly tenfold less than in the wild-type strain and addition of CR did not result in an increase in MpkA phosphorylation levels (Figures 5A and 5B). In agreement with previous results [10], *pkcA* overexpression increases MpkA phosphorylation levels by around 20%, when compared to the wild-type strain, at both the basal level and upon addition of CR (Figures 5A and 5B). In the *alcA::pkcA ΔcnaA* strain the amount of MpkA phosphorylation was higher (18- to 60-fold) than in the wild-type and *ΔcnaA* strains in all conditions tested (Figures 5A and 5B). These results confirm the role of *pkcA* in the CWI pathway in *A. nidulans* and demonstrate that its overexpression can partially suppress the growth defects associated with the Δ*cnaA* strain. In addition, these results also indicate that MpkA is targeted by calcineurin-mediated signaling.

### PkcA overexpression maintains intracellular calcium homeostasis in the absence of CnaA

**Figure 6:**
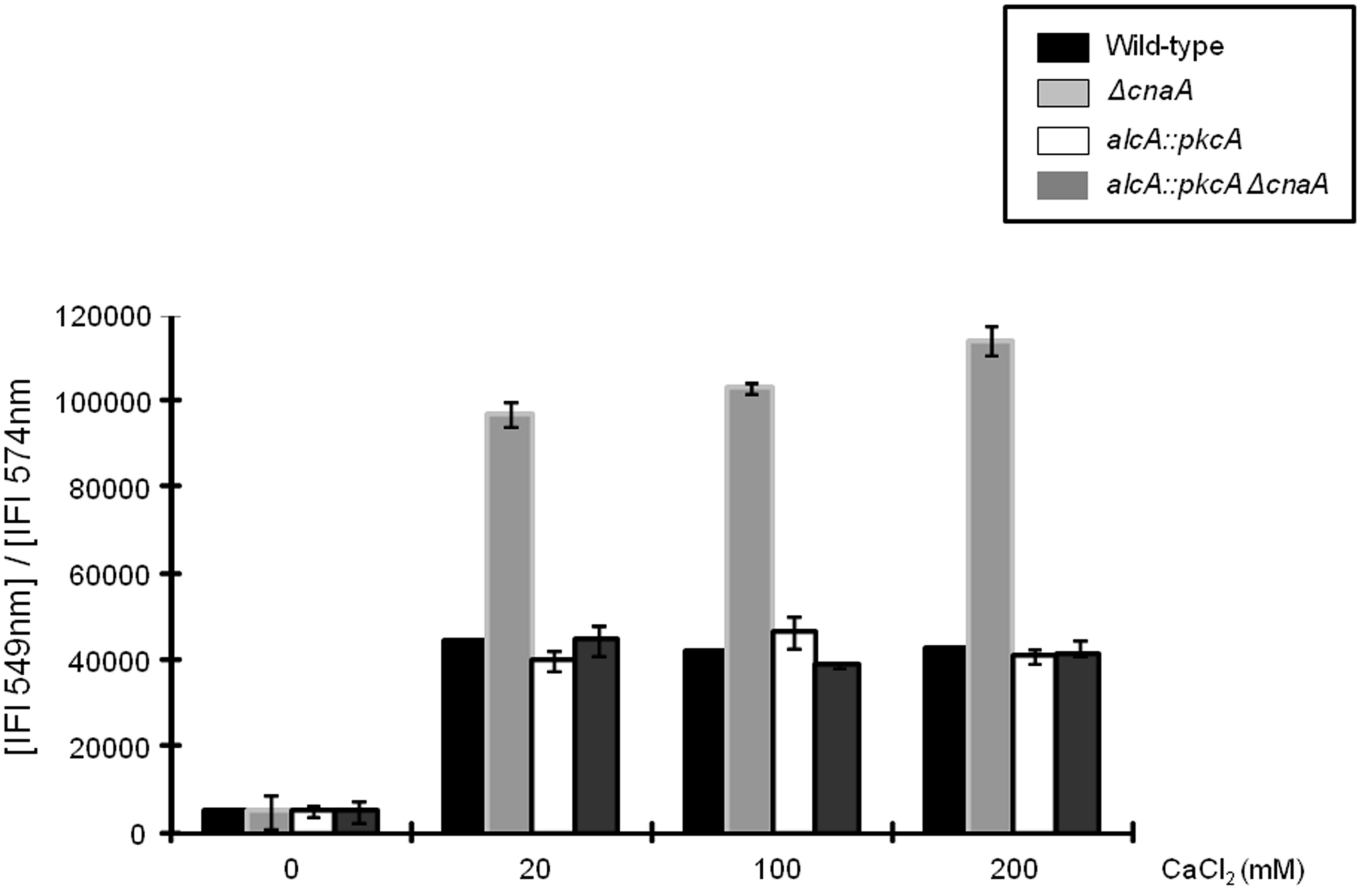
Overexpression of *pkcA* in a Δ*cnaA* background strain enables the cell to return to normal intracellular calcium levels after exposure to high concentrations of CaCl_2_. The wild-type, *alcA*::*pkcA*, Δ*cnaA* and *alcA::pkcA ΔcnaA* strains were grown for 5 hours at 37°C before being exposed to 20 mM, 100 mM and 200 mM of CaCl_2_ for 30 minutes. The fluorophoric calcium chelator Calcium Orange was added then added for 30 minutes and fluorescence was assessed with a fluorometer at 579 nm.

Intracellular calcium homeostasis is mediated through the products of a variety of genes, encoding proteins involved in calcium transport and metabolism, which are regulated by the transcription factor CrzA [29]. Appropriate calcium levels are vital for cell viability, as Ca^2+^ ions sequester phosphate molecules which can cause abnormal intracellular signaling [42]. Hence, to determine if *pkcA* overexpression could restore normal intracellular calcium levels in the absence of CnaA, the wild-type, *alcA::pkcA*, *ΔcnaA* and *alcA::pkcA ΔcnaA* strains were treated with different concentrations of CaCl_2_ for 10 minutes before Calcium Orange fluorophores (COF) were added for 30 min. Calcium orange chelates Ca^2+^ ions and upon binding exhibits increased fluorescence, allowing for the detection of the amount of calcium present (Life Technologies). The wild-type, *alcA::pkcA* and *alcA::pkcA ΔcnaA* strains had reduced intracellular calcium levels after 30 minutes of COF addition (Figure 6). In contrast, the *ΔcnaA* strain was unable to reduce Ca^2+^ levels after 30 minutes of COF addition and had approximately 2.5 times more intracellular Ca^2+^ than the wild-type, *alcA::pkcA* and *alcA::pkcA ΔcnaA* strains (Figure 6). These results indicate that *pkcA* overexpression can contribute to maintaining intracellular Ca^2+^ homeostasis in the absence of CnaA-associated signaling.

### Induction of *alcA*::*pkcA* in the Δ*cnaA* background modulates transcription of calcium controlled processes

**Figure 7:**
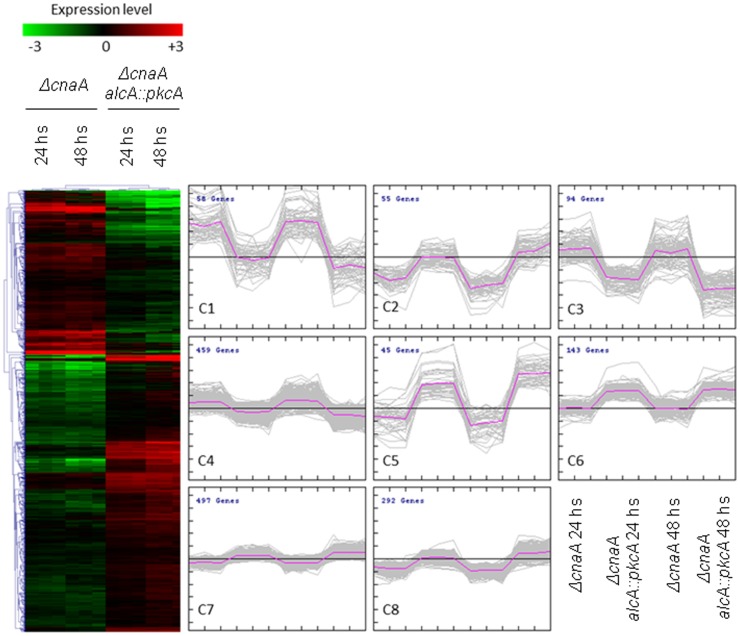
Hierarchal and k-means (KM) clustering of the genes which were differentially (*t*-test *p*<0.001) expressed between the Δ*cna* and *alcA*::*pkcA* Δ*cnaA* strains. Microarrays were performed on RNA extracted from both strains when grown in glucose or glycerol and threonine for 24 and 48 hours.

Microarray hybridizations were utilized to investigate in more detail which genes were being influenced by *pkcA* overexpression in the *alcA*::*pkcA* Δ*cnaA* strain, when compared to the Δ*cnaA* strain (http://www.ncbi.nlm.nih.gov/geo/query/acc.cgi?acc=GSE48535). Both *ΔcnaA* and *alcA*::*pkcA* Δ*cnaA* strains were grown in minimal medium (MM) in the presence of glycerol plus threonine for 24 h and 48 h. The wild-type strain was used as an internal reference for both strains. A total of 1,643 genes were differentially expressed (*t*-test *p*<0.001) between the *ΔcnaA* and *alcA::pkcA* Δ*cnaA* strains. Hierarchical clustering and KMC (K-means clustering) identified 1,032 genes which demonstrated reduced expression in the *ΔcnaA* strain but had expression levels similar to the wild-type strain in the *alcA::pkcA ΔcnaA* strain (Figure 7; clusters 2, 5–8; Table S1). FetGOat analysis of these genes allowed determination of the functions of the proteins they encode and enabled the identification of overrepresented (*p*<0.05) gene ontologies (GO) (Tables S1, S2, S3). As shown in Table 1, the majority of these genes encoded proteins involved in cytoskeletal organization and trafficking, and, in particular, in mitochondria-related functions including oxidative phosphorylation. Genes encoding components of complex III of the respiratory chain and mitochondrial ribosomes were overrepresented within this dataset. The remaining 611 genes had increased expression levels in the Δ*cnaA* when compared to the *alcA*::*pkcA* Δ*cnaA* strain (Figure 7, clusters 1, 3 and 4). However, upon FetGOat analysis, only two GO terms were overrepresented and both described proteasomal components (GO:0000502 and GO:0008540; Table S4).

**Table 1:** Number of genes and the associated functions of the proteins they encode that had restored or elevated expression in the *alcA::pkcA ΔcnaA* strain when compared to the Δ*cnaA* strain.

GO term	Annotation	P-value	sP
**Mitochondrial components, function and energy conversion**			
GO:0006122	mitochondrial electron transport, ubiquinol to cytochrome c	1.12E-04	6
GO:0055114	oxidation-reduction process	5.11E-05	32
GO:0042773	ATP synthesis coupled electron transport	2.99E-05	9
GO:0006626	protein targeting to mitochondrion	9.24E-07	17
GO:0032543	mitochondrial translation	7.89E-04	10
GO:0033617	mitochondrial respiratory chain complex IV assembly	3.85E-04	6
GO:0008535	respiratory chain complex IV assembly	3.85E-04	6
GO:0097034	mitochondrial respiratory chain complex IV biogenesis	3.85E-04	6
GO:0007005	mitochondrion organization	1.40E-20	62
GO:0033108	mitochondrial respiratory chain complex assembly	6.05E-07	10
GO:0007006	mitochondrial membrane organization	2.93E-06	13
GO:0042775	mitochondrial ATP synthesis coupled electron transport	2.99E-05	9
GO:0022904	respiratory electron transport chain	2.99E-05	9
GO:0007007	inner mitochondrial membrane organization	2.99E-05	9
GO:0000002	mitochondrial genome maintenance	6.75E-05	13
GO:0072655	establishment of protein localization in mitochondrion	4.87E-07	18
GO:0022900	electron transport chain	2.99E-05	9
GO:0006839	mitochondrial transport	6.64E-08	22
GO:0045039	protein import into mitochondrial inner membrane	1.80E-03	5
GO:0015980	energy derivation by oxidation of organic compounds	1.28E-07	32
GO:0070585	protein localization in mitochondrion	4.87E-07	18
GO:0045333	cellular respiration	9.65E-12	32
GO:0009060	aerobic respiration	4.64E-12	30
GO:0030150	protein import into mitochondrial matrix	1.72E-05	10
GO:0006119	oxidative phosphorylation	5.19E-04	12
GO:0006099	tricarboxylic acid cycle	7.89E-04	10
**Cytoskeleton-related transport**			
GO:0007018	microtubule-based movement	9.65E-06	11
GO:0072384	organelle transport along microtubule	3.99E-06	11
GO:0031109	microtubule polymerization or depolymerization	2.15E-03	6
GO:0030473	nuclear migration along microtubule	7.16E-05	9
GO:0010970	microtubule-based transport	3.99E-06	11
GO:0007017	microtubule-based process	2.82E-05	22
GO:0030705	cytoskeleton-dependent intracellular transport	9.03E-07	12
GO:0046907	intracellular transport	2.29E-07	91
GO:0017038	protein import	1.26E-07	29
GO:0006810	transport	5.62E-04	136
GO:0071806	protein transmembrane transport	2.23E-05	13
GO:0065002	intracellular protein transmembrane transport	2.23E-05	13
GO:0006886	intracellular protein transport	3.91E-04	41
GO:0015031	protein transport	8.09E-04	41
GO:0007097	nuclear migration	1.20E-05	13
**Cellular organisation**			
GO:0051656	establishment of organelle localization	2.15E-05	23
GO:0034613	cellular protein localization	9.88E-04	46
GO:0006605	protein targeting	9.38E-04	36
GO:0006996	organelle organization	9.06E-08	133
GO:0051647	nucleus localization	1.20E-05	13
GO:0008104	protein localization	1.77E-03	50
GO:0051648	vesicle localization	1.80E-03	5
GO:0051641	cellular localization	6.37E-08	108
GO:0045184	establishment of protein localization	9.46E-04	42
GO:0040023	establishment of nucleus localization	1.20E-05	13
GO:0051234	establishment of localization	1.82E-04	142
GO:0051649	establishment of localization in cell	3.23E-08	99
GO:0033036	macromolecule localization	1.80E-03	61
GO:0051179	localization	1.01E-04	152
GO:0051640	organelle localization	3.10E-05	26
GO:0070727	cellular macromolecule localization	6.01E-04	48
**Metabolism**			
GO:0071840	cellular component organization or biogenesis	4.68E-07	205
GO:0071841	cellular component organization or biogenesis at cellular level	1.49E-07	192
GO:0006364	rRNA processing	2.48E-03	35
GO:0006091	generation of precursor metabolites and energy	6.90E-06	44
GO:0044085	cellular component biogenesis	1.53E-03	92
GO:0016043	cellular component organization	2.78E-05	165
GO:0043623	cellular protein complex assembly	7.16E-05	25
GO:0071842	cellular component organization at cellular level	9.03E-06	146
GO:0006744	ubiquinone biosynthetic process	9.95E-04	6
GO:0006084	acetyl-CoA metabolic process	9.86E-04	11
GO:0046356	acetyl-CoA catabolic process	7.89E-04	10
GO:0006743	ubiquinone metabolic process	9.95E-04	6
GO:0045426	quinone cofactor biosynthetic process	9.95E-04	6
GO:0042375	quinone cofactor metabolic process	9.95E-04	6
**Signal transduction**			
GO:0031684	heterotrimeric G-protein complex cycle	7.04E-04	4

sP is number of genes in each category.

### Overexpression of *pkcA* increases the mitochondria in the *ΔcnaA* genetic background

**Figure 8:**
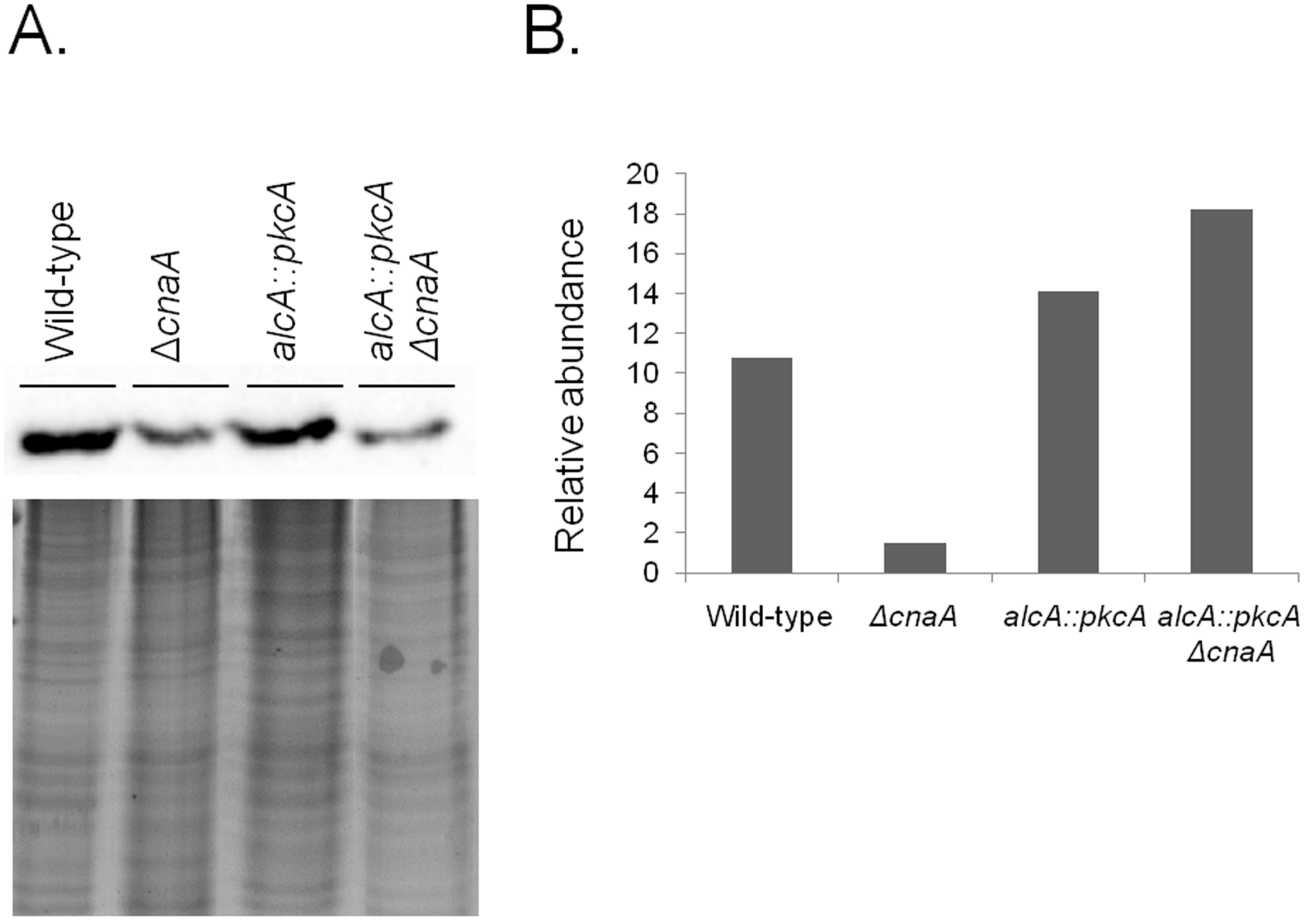
Overexpression of *pkcA* in a Δ*cnaA* background restores cytochrome *c* expression. (A) Western blots of the protein extracts from the wild-type, *ΔcnaA*, *alcA::pkcA* and *alcA::pkcA ΔcnaA* strains when grown in the presence of 2% glycerol plus 100 mM threonine for 16 hours at 37°C. Signal intensities were quantified using the Image J software by dividing the intensity of Western band of the cytochrome *c* by the Coomassie stained protein bands (B). These results are from a single representative experiment from three different repetitions that provided comparable results.

**Figure 9:**
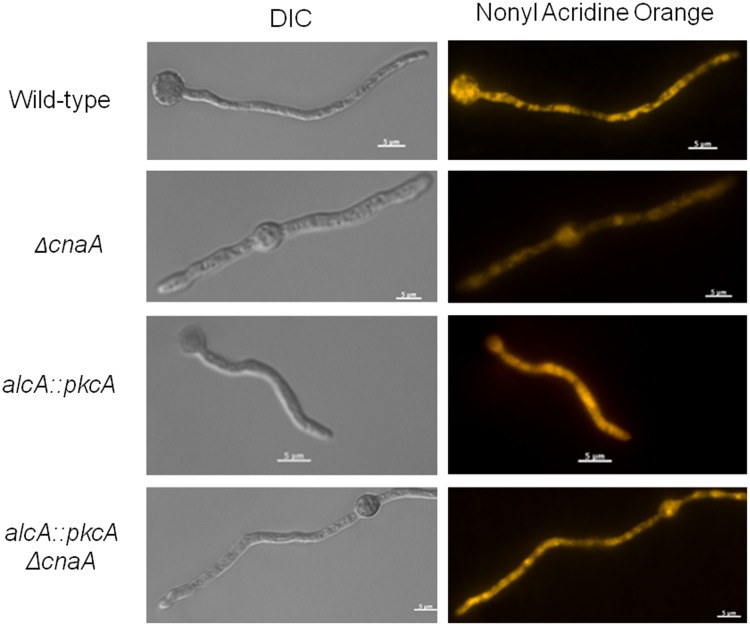
Overexpression of *pkcA* in a Δ*cnaA* background restores mitochondrial number. Wild-type, *ΔcnaA*, *alcA::pkcA* and *alcA::pkcA ΔcnaA* germlings were grown in minimal medium supplemented with 2% glycerol plus 100 mM threonine for 16 hours at 37 C and stained with nonyl acridine orange for 10 minutes at 37°C. Mycelial fluorescence was subsequently assessed under the microscope (scale bars indicate 5 µm).

**Figure 10:**
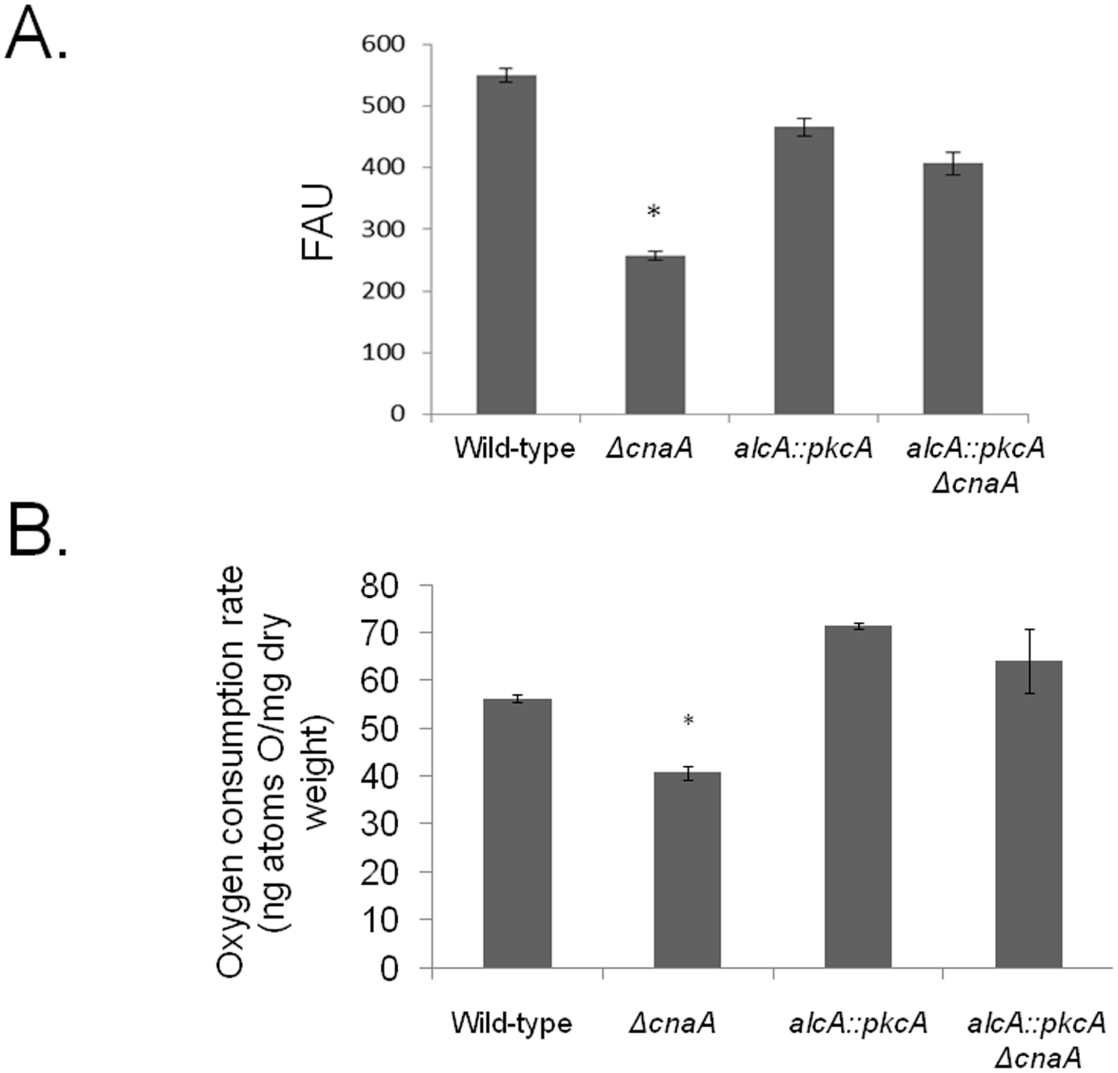
Overexpression of *pkcA* in a Δ*cnaA* background restores oxygen consumption. (A) Wild-type, *ΔcnaA*, *alcA::pkcA* and *alcA::pkcA ΔcnaA* germlings were grown in minimal medium supplemented with 2% glycerol plus 100 mM threonine for 16 hours at 37 C and stained with nonyl acridine orange for 10 minutes at 37°C. Mycelial fluorescence was subsequently assessed under the microscope (scale bars indicate 5 µm). Flow cytometric analyses (FCA) is shown. The results are expressed as mean ± SD (standard deviation) of three independent biological replicates and were considered statistically different (*) when *p*<0.05. P-values were determined by a Student *t* test using GraphPad Prism software version 5 (GraphPad Software). DIC, differential interference contrast; FAU, fluorescent arbitrary units. (B) Oxygen consumption rates of the wild-type, *ΔcnaA*, *alcA::pkcA and alcA::pkcA ΔcnaA* strains when grown in minimal medium supplemented with 2% glycerol plus 100 mM threonine for 16 hours at 37°C.

The microarray data showed a difference in the expression of genes which encoded for proteins involved in mitochondrial functions, between the Δ*cnaA* and *alcA*::*pkcA* Δ*cnaA* strains. Subsequently, to validate these results, mitochondrial mass was evaluated. Protein extracts from the wild-type, *ΔcnaA*, *alcA::pkcA* and *alcA::pkcA ΔcnaA* strains, when grown in MM supplemented with glycerol plus threonine, were incubated with anti-cytochrome *c* antibody (Figure 8A). Cytochrome *c* is a small heme protein found within the cristae of the inner mitochondrial membranes and is an essential component of the electron transport chain [43]. The abundance of cytochrome *c* was less in the *ΔcnaA* strain than the wild-type and *alcA::pkcA* strains (Figure 8B). Upon *pkcA* overexpression, in the *alcA::pkcA ΔcnaA* strain, cytochrome *c* levels were approximately ten times that observed in the *ΔcnaA* strain and were also higher than those observed in the wild-type strain (Figure 8B). These results were in agreement with the fluorescent microscopy data and flow cytometric analyses (FCA), where hyphal mitochondria were stained with Nonyl Acridine Orange, observed under the microscope and subsequently counted, revealing a decrease in mitochondrial mass in *ΔcnaA* strain (Figures 9 and 10A). In addition, the rate of oxygen consumption was measured in all strains. The *ΔcnaA* strain consumed approximately 30% less oxygen than the wild-type strain, whereas *pkcA* overexpression in the *alcA*::*pkcA ΔcnaA* strain restored normal levels of oxygen consumption, similar to the wild-type strain (Figure 10B). These data indicate that *pkcA* overexpression restores the number of mitochondria within the fungal cells, allowing normal levels of respiration to take place.

## Discussion

Cellular signaling governs the cell's responses to environmental stimuli, thus allowing an organism to adapt to different conditions. Cells have evolved a complex network of signaling pathways mediated by a great number of proteins, such as protein kinases and phosphatases, which add or remove phosphate groups from target proteins, therefore directing their activity, location and function [4]. In mammalian cells, protein kinase C (Pkc) regulates gene transcription, membranes and cell growth, while being activated upon an increase in cellular Ca^2+^ levels [2]. In fungi, Pkc plays an important role in growth through activating the cell wall integrity (CWI) pathway which is mediated by a MAP (mitogen activated protein) kinase cascade [11] [14]. In *S. cerevisiae*, cell wall stress sensors activate the Rho GTPase Rho1p which in turn activates Pkc1p that then activates the CWI MAP pathway [17]. In the filamentous fungus *A. nidulans*, the mechanisms for PkcA activation remain unknown. An increase in intracellular Ca^2+^ levels activates the calcineurin phosphatase, whose catalytic subunit A, encoded by *cnaA* in *A. nidulans*, modulates the transcription of genes involved in maintaining cellular calcium homeostasis [29] [31]. Although crosstalk between the calcium- and Pkc-mediated pathways appears to take place in *S. cerevisia*e and other filamentous fungi [32], [33], knowledge about the interactions of these signaling pathways is limited in *Aspergillus* species. The genus *Aspergillus* contains many species of medical and industrial importance, thus elucidating signaling pathways involved in growth in these species could be relevant for many biotechnological applications.

The presented study describes a genetic interaction between the CnaA- and PkcA-mediated signaling pathways in *A. nidulans*. Previous investigations showed that *cnaA* is not an essential gene in *A. nidulans* and *A. fumigatus*, but that its deletion caused severe defects in apical extension and polarized growth in both fungi [26] [34]. Accordingly, the *A. nidulans* Δ*cnaA* strain showed reduced growth in solid and liquid media supplemented with different carbon sources. Furthermore, the *cnaA* deletion strain had a different number of septa and aberrant apical growth when compared to the wild-type strain. The intracellular localization of Ca^2+^ gradients participates in the determination of the site of germ tube emergence and maintains the axis of polarized growth [44]. In *A. fumigatus*, calcineurin localizes to the septum and the catalytic activity of calcineurin subunit A is required for maintaining normal hyphal growth [45]. Deletion of *cnaA* in *A. fumigatus* also resulted in abnormal cell wall architecture with decreased β-glucan and increased chitin contents [45]. In other fungi, calcineurin and Pkc were shown to be involved in the regulation of the cell wall constructing enzyme β-glucan synthase [32], [33]. Similar roles for CnaA in cell wall integrity could therefore be considered in *A. nidulans*.

Overexpressing *pkcA* in the Δ*cnaA* genetic background was able to partially rescue the observed growth Δ*cnaA* phenotypes. PkcA is involved in cell wall construction and maintenance through its involvement in the cell wall integrity pathway via MAPK signaling [11] [14]. In agreement, PkcA overexpression in the Δ*cnaA* strain restored MpkA (the CWI pathway MAPK) phosphorylation levels similar to the wild-type strain. Another explanation could be that both PkcA and calcineurin signaling affect cell cycle progression. In *S. cerevisiae*, a role for Pkc1p in G2/M cell cycle transition, separate from its role in the regulation of the MAPK pathway, has been previously suggested [46]. This could coordinate cell wall expansion with cell cycle progression. In *Schizosaccaromyces pombe*, calcineurin is activated by the cell cycle checkpoint kinase Cds1 and subsequently dephosphorylates Tip1, a protein involved in polarity factor transport, resulting in cell cycle progression delay [47]. All these results indicate that the CnaA- and PkcA-activated pathways share common targets, which could include MpkA, and in doing so coordinate the integrity of the cell wall during growth and in response to stress. This is not surprising as both proteins mediate signaling pathways involved in maintaining polarized hyphal growth.

Microarrays were utilized to provide an insight into the transcriptional impact of *pkcA* overexpression in the Δ*cnaA* strain. A comparision of the Δ*cnaA* and Δ*cnaA pkcA* overexpressing strains with the wild-type strain identified 1,643 genes as being differentially regulated between the two mutant strains, with 1,032 genes being down-regulated and 611 genes being up-regulated in the Δ*cnaA* strain when compared to *alcA*::*pkcA* Δ*cnaA* strain. The majority of the up-regulated genes encoded proteasomal components, suggesting an increase in the degradation of misfolded proteins within the Δ*cnaA* strain. Scrimale *et al.* [48] showed that induction of the CWI pathway in S. *cerevisiae* activated the UPR (unfolded protein response). The UPR is activated upon detection of misfolded proteins in the endoplasmic reticulum (ER), resulting from high throughput protein secretion causing cellular secretory stress [49]. Activation of the UPR results in the up-regulation of genes encoding proteins which control many cellular processes such as protein folding, cell wall architecture, protein trafficking, lipid biosynthesis and ERAD (ER-associated protein degradation) [48]. It appears that deletion of *cnaA* causes an increase in cellular stresses which subsequently activate the UPR and increase protein degradation. Indeed, Colabardini *et al.*, [10] have previously shown crosstalk between PkcA and the UPR in *A. nidulans* in response to exposure to farnesol.

The majority of the down-regulated genes in the Δ*cnaA* strain, when compared to the Δ*cnaA pkcA*-overexpressing strain, encoded proteins involved in mitochondrial function and energy conversion, cytoskeleton-related transport, cellular organization and metabolism. Denis and Cyert [50] showed that the C2 domain of Pkc1p in *S. cerevisiae* is responsible for targeting the protein to the mitotic spindle, whereas the HR1 and C1 regulatory motifs ensure localization of Pkc1p to the bud tip and cell periphery. *A. nidulans* PkcA::GFP localizes to hyphal apices and growing septa, as well as to the conidiogenous apices of phialides, indicating a role for PkcA in polarized cell wall growth [5]. Calcineurin or calmodulin, activated through an increase in intracellular Ca^2+^ levels, have been shown to localize at the apex, at the Spitzenkörper or at the septa during polar growth in *A. nidulans* [51], [52] and *A. fumigatus* [45]. The Spitzenkörper is a sub-apical structure which coordinates polar growth that associates with and is surrounded by microtubules and actin filaments which shuttle vesicles carrying calcium (to ensure the tip-associated calcium gradient), signaling modules and cell wall components back and forth to the Spitzenkörper with the aid of motor proteins [53], [54]. The microarray data showed a reduction in the expression of genes encoding cytoskeletal components, cellular organization and transport in the Δ*cnaA* strain, while the overexpression of *pkcA* was able to restore the expression levels of such genes, further re-inforcing the idea of common targets for the PkcA- and CnaA-mediated signaling pathways, regarding hyphal growth.

Furthermore, genes encoding components of mitochondria were also down-regulated in the Δ*cnaA* strain when compared to the *pkcA* overexpressing strain. Further investigation showed that the deletion of calcineurin caused a decrease in mitochondria and respiration; a defect which was rescued through *pkcA* overexpression. Mitochondria sequester Ca^2+^ ions, therefore playing an important role in decreasing intracellular Ca^2+^ levels after it has carried out its signaling function [3]. Ca^2+^ ions can diffuse readily through the outer membrane of the mitochondria and are then transported through the inner membrane into the mitochondrial cytoplasm through highly selective ion channels and transporters [55]. An increase in mitochondrial Ca^2+^ levels boosts ATP production which as a consequence increases the concentration of cellular reactive oxygen species (ROS) which can lead to apoptosis [54]. In the presented study of *A. nidulans*, deletion of calcineurin caused an increase in cytoplasmic calcium levels, while previous work also showed that calcineurin regulates genes, encoding for proteins involved in calcium homeostasis, via the transcription factor CrzA [31]. It is therefore possible that increased cytosolic calcium levels in the Δ*cnaA* strain resulted in an increased uptake of Ca^2+^ ions by the mitochondria, resulting in increased mitochondrial death. Upon overexpressing *pkcA*, mitochondrial number and function was restored. PkcA-mediated signaling is therefore somehow involved in maintaining the mitochondrial copy number. Indeed, overexpression of *pkcA* upon exposure to farnesol, increases cell death through increasing metacaspase activity [10].

In summary, the presented study describes an interaction between calcium and protein kinase C signaling pathways. Deletion of the calcineurin catalytic subunit A caused growth abnormalities, increased cytosolic Ca^2+^ concentrations plus a reduction in mitochondrial number and function. These defects could all be rescued (at least partially) through the overexpression of *pkcA*. This is the first report in *A. nidulans*, which connects the PkcA- and calcineurin-mediated signaling pathways to mitochondrial function. Further work is required to elucidate at which step these pathways are interconnected, PkcA targets and to investigate in more detail how they link to the mitochondria.

## Materials and Methods

### Strains and media

All *A. nidulans* strains used in this study are listed in Table 2. Strains were cultivated at 37°C in either minimal medium [4% (w/v) glucose or 2% (w/v) glycerol or 2% (w/v) glycerol plus 100 mM threonine], 50 ml of a 20× salt solution (120 g/l NaNO_3_, 10.4 g/l KCl, 30 g/l KH_2_PO_4_, 10.4 g/l MgSO_4_), 1 ml of 5× Trace elements (22.0 g/l ZnSO_4_, 11 g/l boric acid, 5 g/l MnCl_2_, 5 g/l FeSO_4_, 1.6 g/l CoCl_2_, 1.6 g/l CuSO_4_, 1.1 g/l (NH_4_)_2_MoO_4_, 50 g/l EDTA)], pH 6.5; or in complete medium [2% (w/v) glucose, 0.5% (w/v) yeast extract, 1 ml of 5× trace elements (same as for the minimal medium)]. Minimal and complete media were supplemented with 0.12% (w/v) of each uridine and uracil, or pyrodoxine (0.5 µg/ml) depending on the genetic background. Solid media was made by adding 2% (w/v) agar to minimal or complete media. Unless otherwise stated, all chemicals were obtained from Sigma Aldrich (St. Louis, MO, USA).

**Table 2:** *A. nidulans* strains used in this work.

Strain	Genotype	Reference
TNO2A3 (wild-type)	*pyroA4*, *pyrG89*, *argB2*, *ΔnKuA::argB*, *cho1*	Nayak et al., 2006 (reference [58])
*alcA::pkcA*	*pyrG89; pyroA4; alcA::pkcA::pyr4; choA1*	Colabardini *et al.*, 2010 (reference [10])
*ΔcnaA*	*pyroA4 pyrG89*, *ΔcnaA::pyro*, *wA3*	Soriani *et al.*, 2008 (reference [34])
*alcA::pkcA ΔcnaA*	*alcA::pkcA ΔcnaA*, *cho1*	This work

### Mycelial staining and microscopy

Coverslips were inoculated with the different strains (see results section) in 5 ml of minimal media at 30°C.for 16 hours. Hyphae were then fixed [3.7% (v/v) formaldehyde, 50 mM sodium phosphate buffer pH 7.0 (NaH_2_PO_4_, Na_2_HPO_4_), 0.2% (v/v) Triton X-100)] for 30 min at room temperature, rinsed with phosphate buffered saline (PBS: 140 mM NaCl, 2 mM KCl, 10 mM NaHPO_4_, 1.8 mM KH_2_PO_4_, pH 7.4) and incubated for 5 minutes in a 100 ng ml^−1^ calcofluor white. Coverslips were again washed with PBS for 10 minutes at room temperature and rinsed with distilled water. FITC-WGA-staining was carried out by incubating coverslips in prewarmed (30°C) media containing 5 mg ml^−1^ FITC-WGA for 5 minutes [56]. Hyphae were fixed and washed as previously described.

Germlings were visualized on a Carl Zeiss (Jena, Germany) AxioObserver.Z1 fluorescent microscope equipped with a 100 W HBO mercury lamp, using a 100× magnification oil immersion objective (EC Plan-Neofluar, NA 1.3). Fluorescent and DIC (differential interference contrast) images were captured with an AxioCam camera (Carl Zeiss) and processed using the AxioVision software version 3.1. Further image processing was performed using Adobe Photoshop 7.0 (Adobe Systems Incorporated, CA).

### RNA extraction

Mycelia were separated from the supernatant using Whatman filter paper. Fungal cell walls were broken through grinding the mycelia under liquid nitrogen and total RNA was extracted using RNeasy Plant Mini Kit (Qiagen) according to manufacturer's instructions. RNA samples were run on a 2.2 M formaldehyde, 1.2% (w/v) agarose gel to check RNA quality and quantified on the NanoDropH 2000 Thermo Scientific (Uniscience).

### Microarray slides construction and gene expression analysis

Gene expression was compared between *A. nidulans* Δ*cnaA* and *alcA*::*pkcA* Δ*cnaA* strains when grown for 24 and 48 hours at 37°C in in minimal media supplemented with 2% glycerol plus 100 mg/ml threonine, before RNA was extracted. Microarray slide design, sample labeling and hybridization were performed as described previously [56].

The extraction of data from the TIFF files, generated through scanning the microarray slides, was performed using the Agilent Feature Extraction (FE) Software version 9.5.3.1 (Agilent Technologies). This software used the linear Lowess algorithm to subtract the background noise and obtain normalized intensity values. Normalized values were uploaded into the software Express Converter (version 2.1, TM4 platform available at http://www.tm4.org/utilities.html), which converts the Agilent file format to the multi-experiment viewer (mev) file format that is compatible with the TM4 software used for microarray analysis (available at http://www.tm4.org/). The mev files were uploaded into the MIDAS software (TM4 platform), where averages for each gene-replicate, from the biological replicates, were generated. Finally, analysis of the mev files was carried out using the TIGR MeV (TM4 platform, Multi Experiment Viewer, available at http://www.tigr.org/software/microarray.shtml) software. Differentially expressed genes were defined as those that had a mean log_2_ expression ratio statistically different from 0, identified through applying the one-class *t*-test (*P*>0.01).

### Western blots

Intracellular proteins were extracted via grinding the mycelia under liquid nitrogen and then re-suspending the mycelial powder in extraction buffer [15 mM p-nitrophenylphosphate, 25 mM tris pH 7.4, 15 mM EGTA (ethylene glycol tetraacetic acid), 15 mM MgCl_2_ and protease inhibitor cocktail (Complete Mini; Roche)] by vortexing for 10 minutes. Samples were centrifuged for 10 minutes (13,000 rpm) and the supernatant collected. Total protein concentrations in the supernatants were determined using the Bio-Rad Protein Assay, according to manufacturer's instructions. 1× sample loading buffer (62.5 mM Tris-HCl pH 6.8, 2% SDS, 10% glycerol, 5% β-mercaptoethanol and 5% bromophenol blue) was added to the samples and which were boiled at 100°C for 3 min. Samples were run on a 12.5% (w/v) SDS-PAGE gel. The gel was blotted onto a nitrocellulose membrane (0.2 mm; Bio-Rad), which was blocked with a 5% (w/v) dried milk and TBS-Tween buffer (10 mM Tris-HCl pH 8.0, 150 mM NaCl, 0.05% Tween 20, pH 7.5) followed by the incubation with a 1∶1500 dilution of the anti-phospho-p44/42 MAPK antibody (Cell Signaling Technology, USA) overnight at 4°C. Primary antibodies were detected using a horseradish peroxidase (HRP)-conjugated second antibody (Kirkegaard and Perry Laboratories) at a 1∶5000 dilution in TBS-T plus 5% skimmed milk powder for 1 h, at room temperature. Chemiluminescence was detected using Super signal West Pico chemioluminescent substrate (Pierce), and signals were recorded using the Hyperfilm ECL (Amersham Biosciences).

### Oxygen uptake measurements

Conidia (1×10^8^) were inoculated in 15 ml of minimal medium supplemented with 2% glycerol plus 100 mM threonine for 5 hours at 37°C, 250 rpm. The germlings were harvested by centrifugation and washed twice with cold distilled water. Germlings were then washed three times with 0.7 mM sorbitol, 10 mM HEPES–KOH, pH 7.2 and subsequently kept on ice. Oxygen uptake was measured with a Clark-type electrode fitted to a Gilson oxygraph 1 (Gilson Medical Electronics, Inc.,2 Middleton, WI) in 1.8 ml of buffer containing 0.7 mM sorbitol, 10 mM HEPES–KOH, pH 7.2, 5 mM MgCl_2_ and 0.5 mM EGTA at 30°C [57]. The initial solubility of oxygen in the reaction buffer was considered to be 445 ng of O_2_ atoms/ml.

### Mitochondrial mass measurements

Conidia from the different strains (1×10^8^ ml^−1^) were incubated in 50 ml liquid minimal media at 37°C on a reciprocal shaker for 6 hours. Subsequently, conidia were washed with PBS and incubated with 5 nM Nonyl Acridine Orange (NAO; Invitrogen) diluted in PBS plus 5% fetal bovine serum (FBS) for 10 minutes at 37°C. Stained conidia were washed with PBS, resuspended in PBS plus 5% FBS, and then analyzed by flow cytometry. Propidium iodide (0.5 µM) staining of the nucleus was utilized to exclude dead cells. Flow cytometry was analyzed by guava Easycity 8 HH (Millipore) using 10,000 acquisitions for each analysis.

For cytochrome *c* measurements, 2×10^8^ conidia/ml of each strain were inoculated in minimal media for 16 hours at 37°C. After this period, the cultures were filtered and the mycelia washed with 100 ml H_2_O, immediately frozen in liquid nitrogen and grinded. For every 0.1 g of dry weight, 1.3 ml HB buffer (150 mM NaCl, 30 mM KCl, 10 mM Na_2_HPO_4_, pH 7.0) was added. The suspension was centrifuged at 21,000 *g* at 4°C for 30 minutes. The supernatant was removed and centrifuged again for 10 minutes under the same conditions. After determining protein concentration, samples were prepared for SDS-PAGEas described above. Thirty micrograms of total protein from each sample were loaded into each lane of a 15% SDS-PAGE gel. After separation of the proteins, the gel was blotted onto a pure nitrocellulose membrane (0.2 µm; Bio-Rad) and after being blocked in 5% (w/v) dried milk in TBSTween, the membrane was probed with the rabbit anti-cyc1 antibody (CNAT against native cytochrome *c* from yeast; Sigma) at a 1∶200 dilution in TBS-Tween for 1 hour at room temperature. The membrane was washed four times with TBS-Tween for 5 minutes and then incubated with a 1∶5000 dilution of goat anti-rabbit IgG peroxidase-labeled (KPL) antibody for 1 hour. After being washed, the blot was developed by use of the SuperSignal Ultra chemiluminescence detection system (Pierce) and recorded by the use of Hyperfilm ECL (Amersham Biosciences).

### Determination of intracellular calcium levels

To determine intracellular Ca^2+^ levels, wild-type, Δ*cnaA*, *alcA*::*pkcA* and *alcA*::*pkcA* Δ*cnaA* strains were first grown for 5 hours in minimal medium supplemented with 2% (w/v) glycerol plus 100 mM threonine at 37°C. Strains were then treated with 20 mM, 100 mM and 200 mM CaCl_2_ for 10 minutes. Mycelia were then washed 3 times with PBS and incubated with 1 µM Calcium Orange fluorophore at 37°C for 30 minutes. Mycelia were washed again and fluorescence was measured using a fluorometer at 549 nm excitation and 579 nm emission, according to manufacturer's instructions. The experiment was normalized by counting the number of cells in a Neubauer chamber.

## Supporting Information

Table S1 **FetGOat analysis for genes with reduced mRNA accumulation in** ***A. nidulans ΔcnaA*** **(similar to the wild-type strain) when compared to the** ***alcA::pkcA ΔcnaA*** **strain.**
(XLSX)

Table S2 **FetGOat analysis for genes with reduced mRNA accumulation in** ***A. nidulans alcA::pkcA ΔcnaA*** **(similar to the wild-type strain) when compared to the** ***ΔcnaA*** **strain.**
(XLSX)

Table S3 **FetGOat analysis for genes with increased mRNA accumulation in** ***A. nidulans alcA::pkcA ΔcnaA*** **(similar to the wild-type strain) when compared to the** ***ΔcnaA*** **strain.**
(XLSX)

Table S4 **FetGOat analysis for genes with increased mRNA accumulation in** ***A. nidulans ΔcnaA*** **when compared to the** ***alcA::pkcA ΔcnaA*** **strain.**
(XLSX)
