# Correction: Protein Kinase C Overexpression Suppresses Calcineurin-Associated Defects in *Aspergillus nidulans* and Is Involved in Mitochondrial Function

**DOI:** 10.1371/journal.pone.0113985

**Published:** 2014-11-12

**Authors:** The PLOS ONE Staff

The sixth author’s name is spelled incorrectly. The correct name is: Marcia Regina von Zeska Kress.
